# Aggressive behavior, boredom, and protective factors among college students during closed-off management of the COVID-19 pandemic in China

**DOI:** 10.3389/fpsyg.2022.1012536

**Published:** 2022-12-16

**Authors:** Yujie Li, Xiaoyi Chu

**Affiliations:** Department of Health Management, Shandong Drug and Food Vocational College, Weihai, China

**Keywords:** COVID-19, boredom, cognitive flexibility, aggression, moderation

## Abstract

**Background:**

Chinese colleges have implemented strict closed-off management in response to the outbreak of a new variant of the new coronavirus, Omicron. But such management measures may lead to more aggressive behavior. The study aimed to determine the associations between boredom and aggressive behavior with aggression and to examine the impact of boredom on aggression through the moderating role of cognitive flexibility.

**Methods:**

The Multidimensional State Boredom Scale, the Reactive–Proactive Aggression Questionnaire, and the Cognitive Flexibility Inventory were applied to a sample of 719 college students who were in a closed-off management environment.

**Results:**

For individuals with high cognitive flexibility, the relationship between state boredom and proactive aggression was not significant. The relationship between state boredom and proactive aggression was significantly positively correlated for individuals with low cognitive flexibility, especially low substitutability. Cognitive flexibility has no significant moderating effect on the relationship between state boredom and reactive aggression.

**Conclusion:**

The findings highlighted the importance of boredom as a potential risk factor for aggression, while cognitive flexibility appears as a potential protective factor.

## Introduction

COVID-19, a novel coronavirus disease, has caused numerous infections worldwide. To break the transmission link of the virus and curb the outbreak of the COVID-19 pandemic, the Chinese government has taken aggressive public health monitoring and interventions, such as mass nucleic acid testing, contact tracing, travel restrictions, and avoiding crowd gathering (Cheng et al., 2021; Hu et al., 2021; Wan et al., 2022). As the Omicron variant has caused COVID-19 resurgences in many places, in cities with severe epidemics, primary and middle schools have to be closed and converted to online teaching, and colleges have implemented relatively closed-off management. Except for necessary medical treatment, college students are not permitted to leave campus without special circumstances, in a bid to reduce the likelihood of COVID-19 (Roberton et al., 2012; Sun et al., 2021; Zhang et al., 2021; Tu et al., 2022; Zhang and Zhu, 2022).

Such strict quarantine and restrictive policies have greatly relieved the pressure on the healthcare system and played a role in keeping infection and death rates low (Fu et al., 2021; Ge et al., 2021; Bo et al., 2022). These policies, however, also affect normal study, socialization, and life, potentially leading to psychological and behavioral problems for college students (Copeland et al., 2021; Li et al., 2021a, b; Baleanu et al., 2022).

### Aggression

In general, aggression is defined as behavior with the immediate intention of harming another individual. Moreover, the perpetrator must believe that the behavior will cause harm to the target as well as the target must be motivated to avoid the behavior (Anderson and Bushman, 2002). Reactive aggression occurs in response to a real or perceived threat, whereas proactive aggression occurs in order to accomplish a specific goal(Miller and Lynam, 2006; Romero-Martínez et al., 2022). During the COVID-19 pandemic, many young people have been directly or indirectly exposed to violence and aggression during the pandemic (Field, 2021; Bera et al., 2022). Compared with people who were not under stay-at-home restrictions, individuals who were under lockdown status were more likely to be depressed, face more domestic violence risks (Humphreys et al., 2020; Mazza et al., 2020). A significant number of students showed more and more destructive and aggressive behavior (Pfefferbaum and North, 2020; Killgore et al., 2021; Ye et al., 2021). Not only that, the content of aggressive behavior also appears in dreams (Kilius et al., 2021). Researchers have examined changes in aggressive behavior before and after the epidemic, and found a rise in cyberbullying behaviors, physical aggression, verbal aggression (Barlett et al., 2021; Wang et al., 2022).

Various empirical studies show that the emotion regulation motivation may play an important role in aggression (Bushman et al., 2001; Roberton et al., 2012; DeWall et al., 2016; Chester et al., 2019). There is preliminary evidence in the literature that indicates that under-regulation of emotion is likely to be associated with aggressive behavior. The presence of uncomfortable emotions, which an individual cannot otherwise manage, is likely to increase his or her willingness to act aggressively (Roberton et al., 2012). In some situations, aggression allows the individual to externalize their internal emotional state and regulate others’ behavior. A person may engage in aggressive behavior in the hope that it will make them feel better (Bushman et al., 2001).They believe that aggressive behavior could facilitates the control of emotional experiences, alleviates discomfort, and contributes to the achievement of goals (Bushman et al., 2001; Baumeister et al., 2007; Pfattheicher et al., 2021b).

### Boredom and aggression

Boredom is the adverse experience of wanting, but being unable, to engage in stimulating and satisfying activity (Eastwood et al., 2012; Van Tilburg and Igou, 2012; Elpidorou, 2018; Westgate and Wilson, 2018). There are two types of boredom: state boredom (an emotion that appears in a specific setting) and trait boredom (an individual*’*s proneness to experience feelings of disinterest). According to the Meaning and Attention Components (MAC) model of boredom, boredom emerges when the task have little meaning or under stimulating (Mercer-Lynn et al., 2014; Westgate and Wilson, 2018; Liang et al., 2020). During the COVID-19, the reduced autonomy or perceived limitations in environment leads to a lower degree of individual arousal, cognitive resources may not optimally used (Liang et al., 2020; Weybright et al., 2022). Such monotonous and constrained quarantine environment is more likely to increase the risk that individuals will experience state boredom (Homel et al., 1992; Rupp and Vodanovich, 1997; Dahlen et al., 2004; Elpidorou, 2018). In order to fight it, individuals have to change their behavioral or cognitive patterns (Nett et al., 2011).

Findings from the psychological and neural sciences have shown that aggressive behavior can indeed reduce boredom and bring positive feelings to some extent (Raine et al., 2006). Such aggressive pleasures may have evolved from predatory behaviors that were later rewarded with reproductive benefits (Griskevicius et al., 2009; Chester, 2017; LIU et al., 2022).Various studies have shown that boredom is associated with aggressive behavior, such as dangerous driving (Dahlen et al., 2005), self-harm (Chapman and Dixon-Gordon, 2007; Nederkoorn et al., 2016), school bullying, and abusive behavior (Pfattheicher et al., 2021a), etc. In an empirical study, Homel, Tomsen, and Thommeny examined the relationship between boredom proneness and aggressive behavior. They founded that boredom affected adolescents’ aggressive behaviors such as public violence and alcohol-related aggression (Homel et al., 1992). This view was confirmed by research by Rupp and Vodanovich, who found that a high total boredom score was positively correlated with aggression scores, significantly predicting the expression of aggressive behavior (Rupp and Vodanovich, 1997). Vodanovich concluded from a review of previous studies that individuals with high boredom have higher levels of aggression and are prone to bad social behaviors such as alcoholism, drug use, and violence (Vodanovich, 2003). People may even regulate their boredom through exposure to violent contents and through mediated aggression (Vandebosch and Poels, 2021).

### Moderating role of cognitive flexibility

It is worth noting that the current emotional state cannot determine whether an individual engages in aggression (Rupp and Vodanovich, 1997; Dahlen et al., 2004). Not all of us fought boredom with aggressive behavior during the COVID-19 pandemic. Both person factors (e.g., personality traits) as well as situational factors (e.g., aggressive cues) affect an individual*’*s readiness to engage in aggression (Dahlen et al., 2004). Recent research has found that anticipating the emotions and the consequences of actions has a major impact on behavior (Chester et al., 2019). If individuals believe that aggression worsens their emotional state, their aggressive behavior will not increase or even decrease under negative emotions (Bushman et al., 1999).

Cognitive flexibility plays a key role in reappraising situations (Dennis and Vander Wal, 2010; Inozu et al., 2022). It refers to people*’*s mental ability to switch cognitive sets to adapt to changing environmental stimuli (Martin and Rubin, 1994; Dennis and Vander Wal, 2010). Individuals with high cognitive flexibility solve problem through more constructive and adaptive cognition (e.g., focus on problem coping, focus on the positive, seek social support; Rende, 2000; Kalia et al., 2019). They perceive difficult situations as controllable and generate multiple alternative explanations for life events (Dennis and Vander Wal, 2010). Cognitive flexibility has been shown to be a protective factor against external and internal stress (Koesten et al., 2009; Dennis and Vander Wal, 2010; Murphy et al., 2012; Sağar, 2021). Rather than ruminate on the perceive inability to problem solve, it can motivate individuals to generate multiple alternative solutions (Dennis and Vander Wal, 2010). Individuals with cognitive flexibility may be able to reframe their understanding of global pandemics. It may enable them to reconsider behaviors that would mitigate their risk in a challenging environment (Bonanno and Burton, 2013).

In fact, people*’*s attempts to regulate their emotion through aggression may be risky and counterproductive. Due to the fact that aggression can cause more physical and psychological harm to both parties, pleasure may be short-lived and soon replaced by discomfort. In addition, cultural values and beliefs may inhibit or encourage people*’*s expressions of aggression (Bond, 2004). In the perspective of an individualist, aggression can be viewed as a method for achieving self-reliance and winning competitions, whereas in a collectivist perspective, aggression leads to an erosion of interpersonal relations and group harmony (Li et al., 2010). It appears that aggression may not be the most effective means of regulating emotions. By extending previous research on the relationship between boredom and aggressive behavior, exploring how the cognitive flexibility influence the decision-making, a deeper understanding of the mechanisms can be gained. We could provide individuals with better options for regulating emotions.

### The current study

In the present study, we sought to determine whether state boredom is associated with two forms of aggressive behaviors (proactive aggression and reactive aggression). In addition, cognitive flexibility was divided into two facets (control and alternative), enabling a more nuanced distinction between the variables. Based on a hierarchical regression model, we examined whether cognitive flexibility moderates the relationship between state boredom and aggressive behavior. We hypothesized that there would be a significant positive relationship between the state boredom and aggressive behavior. Moreover, cognitive flexibility would show a significant negative relationship with aggressive behavior. Finally, cognitive flexibility would moderate the relationship between state boredom and aggressive behavior.

## Materials and methods

### Participants

719 Chinese participants (356 male; age range 18–22; Mage = 20.56, SDage = 2.33) were recruited from a college in Shandong province in China to participate in this study in April 2022. As the Omicron variant has caused COVID-19 resurgences, these participants have been under the strict closed-off management for nearly 2 months.

Investigators explained the study to all participants before collecting any data. Each participant provided written consent prior to the beginning of the study, which was approved by the researchers’ University Ethical Advisory Committee. All participants were required to indicate their demographic information and complete three questionnaires. They were tested independently, lasting approximately 25 min, and all received same research credit in exchange for participation. Researchers encouraged students to respond as truthfully as they could, highlighting that their answers would be kept confidential.

### Measures

#### State boredom

The Multidimensional State Boredom Scale (MSBS) is a self-reported 29-item scale developed by Fahlman et al. (2013). We used the Chinese version of Liu et al. (2013), which was revised according to Chinese cultural background. In accordance with both theoretical and empirical definitions of boredom, the boredom scale identifies five factors: disengagement, high arousal, low arousal, inattention and time perception. Using Likert 7 grade score (completely disagree–completely agree, in turn recorded as 1~7 points), the higher total 24 items score represents the higher levels of state boredom. In previous studies, the scale has shown good reliability and validity (Ng et al., 2015; Zhao et al., 2016; Liang et al., 2020). In this study, the Cronbach’s alpha was 0.912.

#### Cognitive flexibility

The cognitive flexibility inventory (CFI) is a brief self-reported cognitive flexibility measurement tool developed by Dennis and Vander Wal (2010). The CFI measures aspects of cognitive flexibility that enable individuals to respond adaptively to stressful life events. We used the Chinese version of Wang et al. (2016), which was revised according to Chinese expression habit. The scale consists of two dimensions (Alternatives and Control). The items use a 7-point Likert rating system with response options ranging from 1 (completely disagree) to 7 (completely agree). There are 13 items in the Alternatives subscale, which measures the ability of individuals to generate alternative explanations for occurrences and alternative solutions to problems. The Control subscale consists of 7 items, which measure an individual*’*s tendency to perceive difficult situations as controllable. Items were reverse scored when necessary and summed. The higher total score represents the higher levels. In previous studies, the scale has shown good reliability and validity (Yu et al., 2020; Zhou et al., 2020; Zou et al., 2020). In this study, the Cronbach’s alpha was 0.856.

#### Aggressive behavior

The Reactive–Proactive Aggression Questionnaire (RPQ) is a brief is a self-report questionnaire designed to assess reactive and proactive aggression in adolescent (Raine et al., 2006). We used the Chinese version of Zhang et al. (2014) which was revised according to Chinese cultural background. The scale consists of two dimensions (proactive aggression and reactive aggression). It has a 6-point Likert rating system with response options ranging from 1 (not at all characteristic of me) to 6 (entirely characteristic of me), the higher total items score represents the higher levels of aggressive behavior. In previous studies, the scale has shown good reliability and validity (Fossati et al., 2009; Fung et al., 2009; Pechorro et al., 2017). In this study, the Cronbach’s alpha was 0.877.

### Statistical analyses

SPSS 24.0 was used to process the data for this study. The first step was analyzing whether the data had a common method bias using Harman’s single-factor test (Pm, 2003). In the second step, descriptive statistics and Pearson bivariate correlations were used to analyze the scores from the three questionnaires. As a final step, the moderation model was tested using the SPSS macro PROCESS (model 1) introduced by Hayes et al. (2017). The age and gender were entered as covariant into the moderation model. For the significant effects, pick-a-point approximation was used to interpret the results.

## Results

### Common method biases

By using factor analysis, a common variance analysis was applied to the three questionnaires. As a result of Bartlett’s test of spherical, the chi-square reached significance. A total of 15 eigenvalues greater than one were extracted after principal component analysis. There was a first factor that explained 13.69% of the variance, which was less than the 40% required by the critical standard (Pm, 2003). It appears that common method bias is not a major concern based on these results.

### Descriptive and bivariate correlations analysis

Table 1 provides descriptive statistics and a correlation matrix for state boredom, aggressive behavior and its sub-dimensions (proactive aggression and reactive aggression), and cognitive flexibility and its sub-dimensions (alternatives and control). Bivariate correlation analysis revealed a negative correlation between aggression and cognitive flexibility (*r* = −0.085, *p* < 0.05) and a positive correlation between aggression and boredom (*r* = 0.145, *p* < 0.01). Moreover, Proactive aggressive behavior score was negatively correlated with cognitive flexibility (*r* = −0.114, *p* < 0.01).

**Table 1:** Descriptive statistics and results of correlational analysis.

Variables	Mean	SD	Median	Mode	1	2	3	4	5	6
1 Boredom	89.22	33.03	91	96	1					
2 Cognitive flexibility	73.28	31.23	71	65	0.019	1				
3 Alternatives	50.58	21.79	49	46	0.016	0.866^**^	1			
4 Control	22.7	11.63	21	19	0.022	0.875^**^	0.721^**^	1		
5 Aggressive behavior	54.99	20.58	55	50	0.145^**^	−0.085^*^	−0.116^**^	−0.009	1	
6 Proactive aggression	24.92	13.44	21	12	0.158	−0.114^**^	−0.167^**^	0.007	0.614^**^	1
7 Reactive aggression	30.06	16.25	27	10	0.052	−0.013	−0.009	−0.017	0.758^**^	−0.049

* *p* < 0.05.

** *p* < 0.01.

### Moderation effect of cognitive flexibility on the relationship between boredom and aggressive behavior

The results of the moderation analysis with selected aggressive behavior (and its components) as the dependent variable, boredom as an independent variable, and cognitive flexibility as a moderator are presented in Table 2.

**Table 2:** Results of moderation analysis with the aggressive behavior, proactive aggression, and reactive aggression as dependent variables, boredom as the independent variable, and cognitive flexibility as the moderator.

Interaction effect	Coefficient	*SE*	*t*	*P*
**Aggressive behavior as dependent variable**				
Boredom × *CF*	−0.085	0.039	−2.176	0.03
**Proactive aggression as dependent variable**				
Boredom × *CF*	−0.101	0.039	−2.588	0.01
Boredom × A	−0.101	0.038	−2.646	0.008
Boredom × C	−0.072	0.039	−1.853	0.064
**Reactive aggression as dependent variable**				
Boredom × *CF*	−0.025	0.04	−0.619	0.536
Boredom × A	−0.024	0.04	−0.595	0.552
Boredom × C	−0.019	0.039	−0.473	0.636

CF, Cognitive Flexibility; A, Alternatives; C, Control.

**Figure 1:**
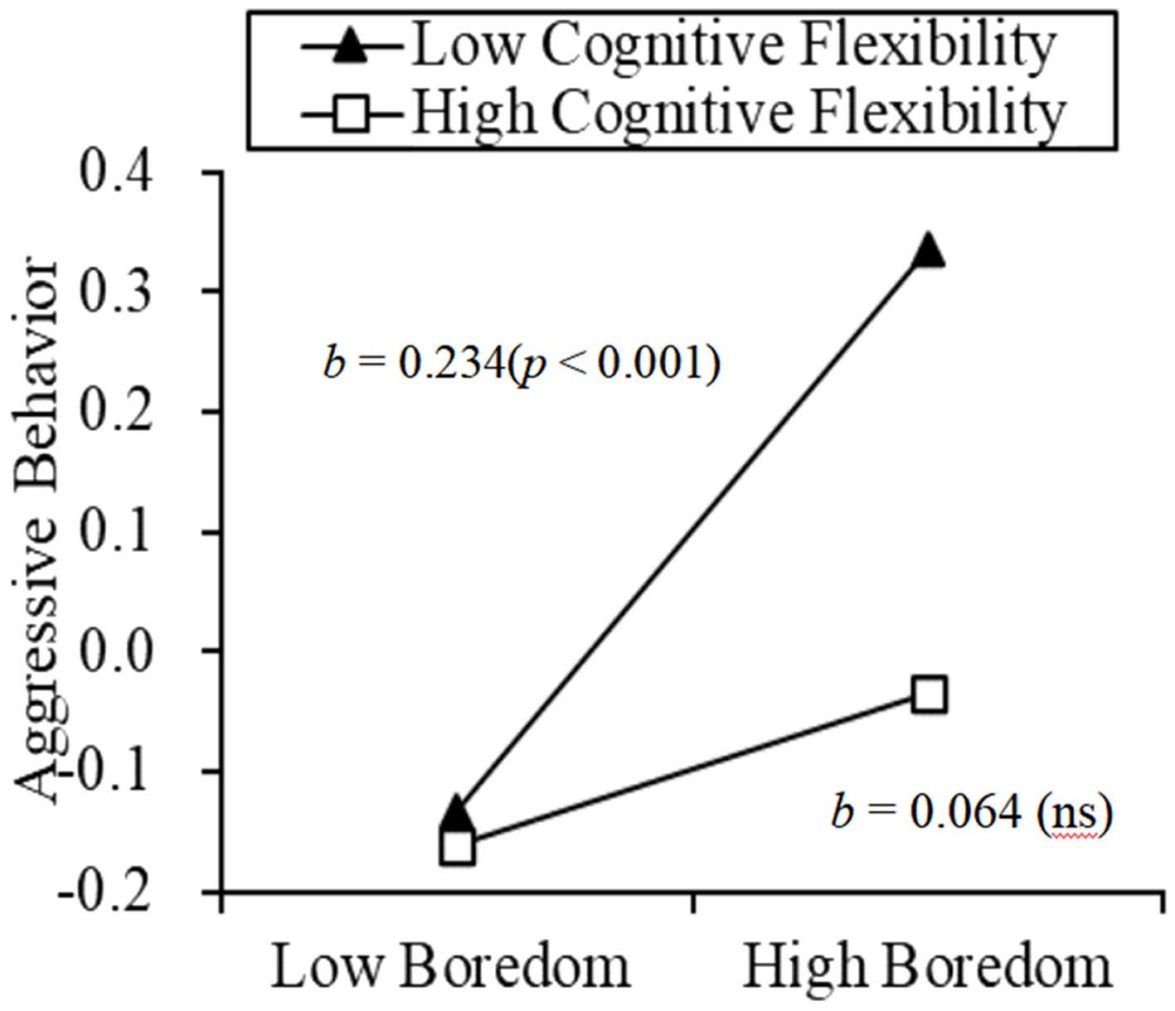
Moderation effect of cognitive flexibility on the relationship between boredom and aggressive behavior.

The results show that cognitive flexibility moderated the relationship between boredom and aggressive behavior (*β* = −0.085, *p* < 0.05). Results of a simple slope test further revealed that, for individuals with low cognitive flexibility, state boredom could positively predict aggressive behavior (*β_simple_* = 0.234, *p* < 0.001). For individuals with high cognitive flexibility, the relationship between state boredom and aggressive behavior was not significant (*β_simple_* = 0.064, *p* = 0.228; see Figure 1).

**Figure 2:**
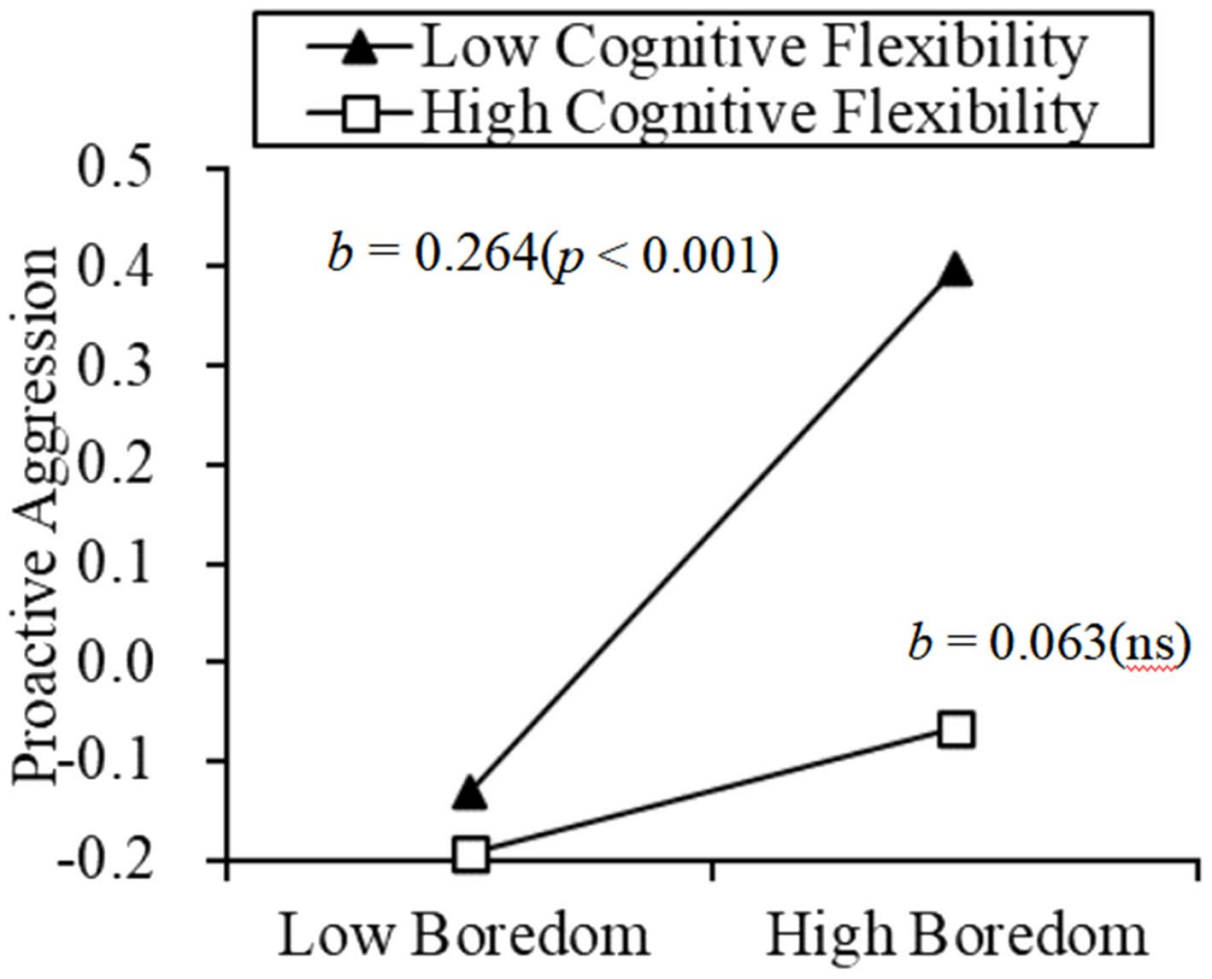
Moderation effect of cognitive flexibility on the relationship between boredom and proactive aggression.

**Figure 3:**
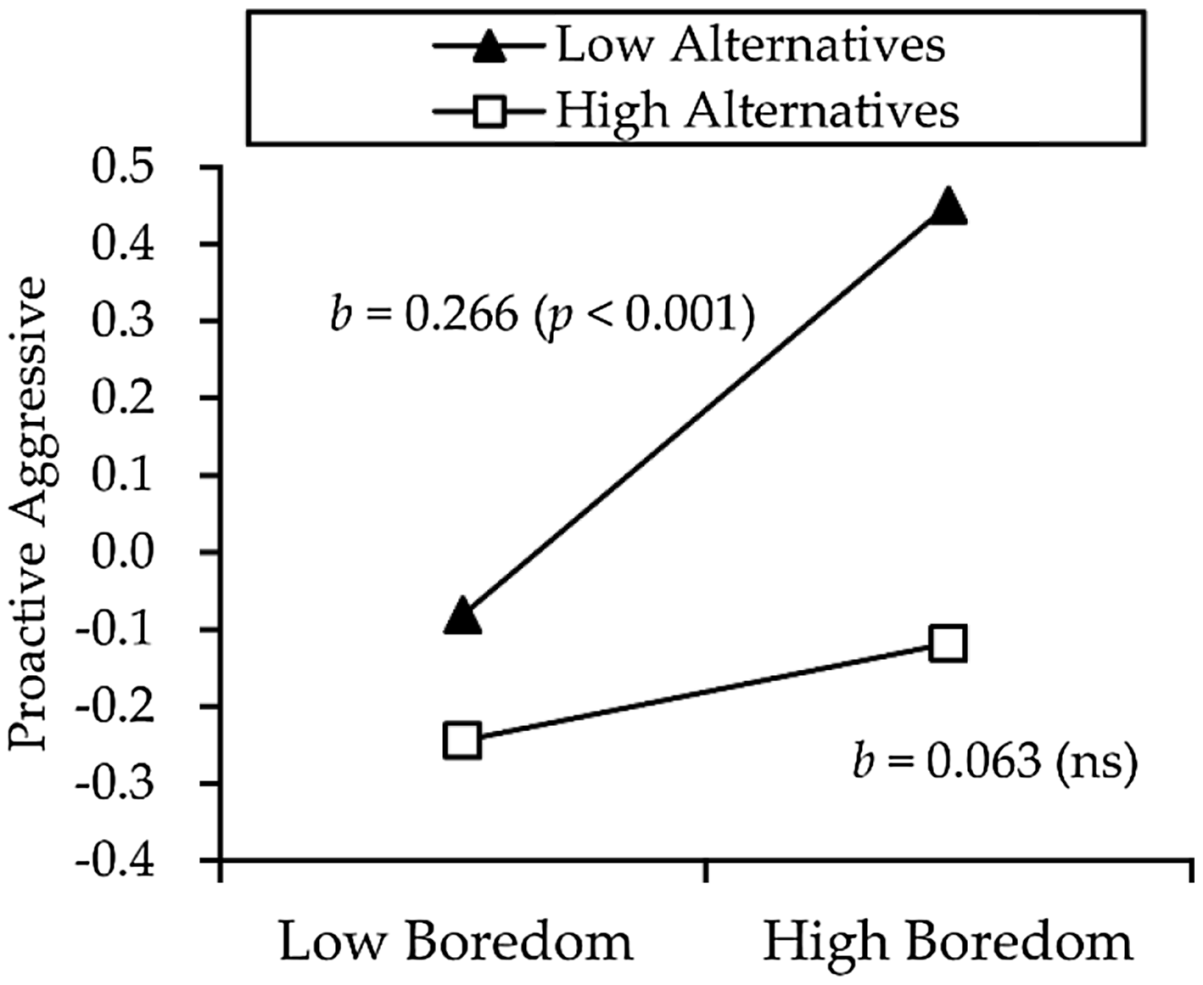
Moderation effect of alternatives on the relationship between boredom and proactive aggression.

Further, the various components of aggressive behavior were used as dependent variables. Cognitive flexibility and its two subcomponents were used as moderators, respectively. The results are as follows: cognitive flexibility moderated the relationship between boredom and proactive aggression (*β* = −0.101, *p* < 0.05). Results of a simple slope test further revealed that, for individuals with low cognitive flexibility, state boredom could positively predict proactive aggression (*β_simple_* = 0.264, *p* < 0.001). For individuals with high cognitive flexibility, the relationship between state boredom and proactive aggression was not significant (*β_simple_* = 0.063, *p* = 0.232; see Figure 2). Moreover, alternatives moderated the relationship between boredom and proactive aggression (*β* = −0.101, *p* < 0.01). Simple slope test revealed that, for individuals with low alternatives, state boredom could positively predict proactive aggression (*β_simple_* = 0.266, *p* < 0.001). For individuals with high alternatives, the relationship between state boredom and proactive aggression was not significant (*β_simple_* = 0.063, *p* = 0.224; see Figure 3). Control has no significant effect on the relationship between state boredom and proactive aggression (*β* = −0.072, *p* = 0.064).

**Figure 4:**
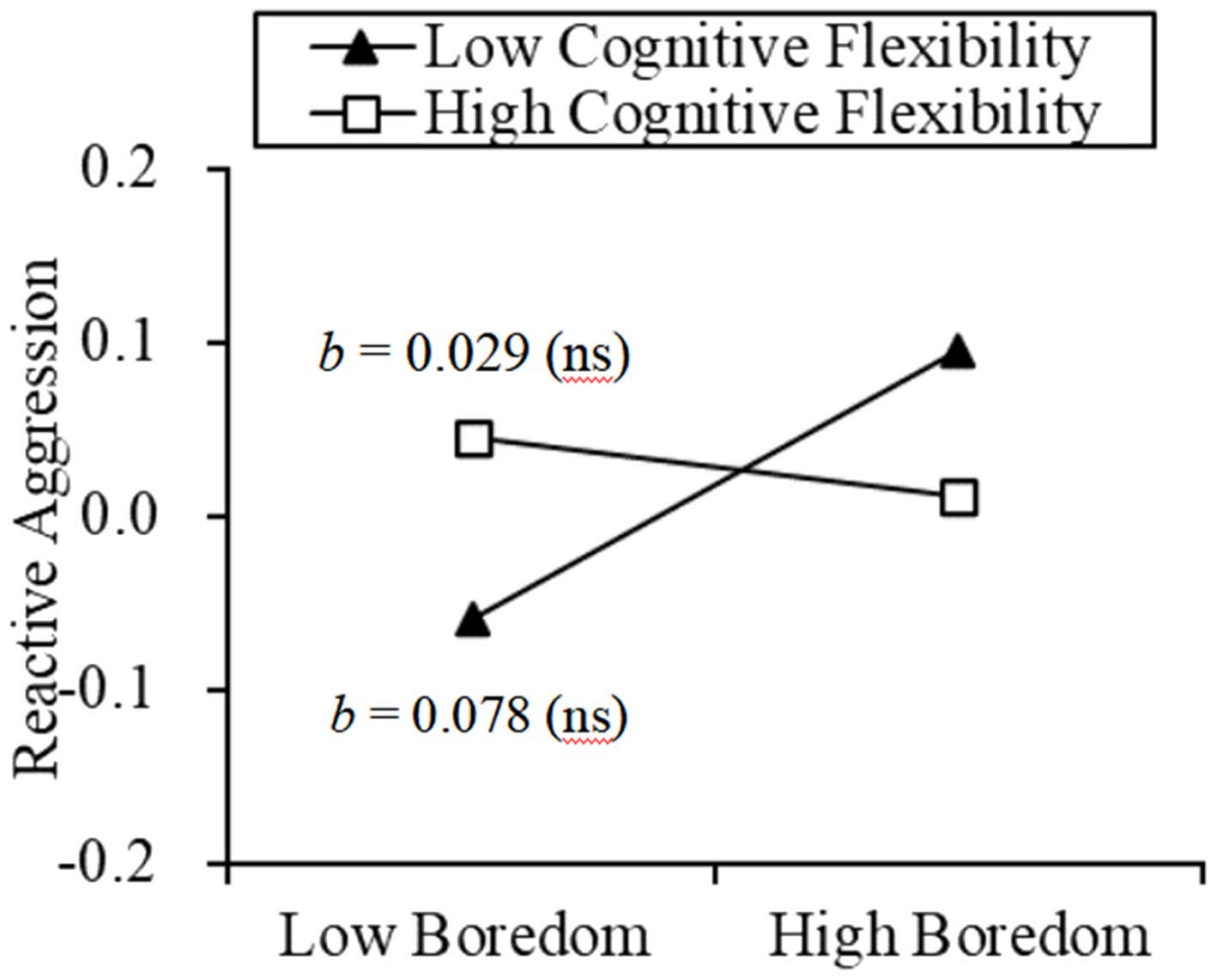
Moderation effect of cognitive flexibility on the relationship between boredom and reactive aggression.

For reactive aggression, cognitive flexibility has no significant effect on the relationship between state boredom and reactive aggression (*β* = −0.025, *p* = 0.536; see Figure 4). Alternatives and control also have no significant effect on the relationship between state boredom and reactive aggression (*β* = −0.024, *p* = 0.552; *β* = −0.019, *p* = 0.636).

## Discussion

### The relationship between boredom and aggression

When the environment is monotonous, repetitive, boring, etc., or the environment does not match the internal standards, it is easy to induce the individual*’*s state boredom. Due to the new coronavirus epidemic caused by the “Omicron” variant, college students are facing more inconvenience and restrictions in their lives, which significantly increased the boredom level (Chao et al., 2020). Individuals will adopt coping strategies when their environments cannot be exited or changed. The purpose of this study was to examine the relationship between boredom and aggressive behaviors (proactive aggression and reactive aggression) during close-off management.

As a coping strategy for boredom, there was no significant positive association between the two forms of aggressive behaviors and boredom. The results of this study are in line with previous research on coping strategies and boredom (Droit-Volet et al., 2020; Gazmer et al., 2020; Liang et al., 2020; Donati et al., 2022). In light of this, aggression may not be a meaningful and satisfying alternative target activity for everyone as a means to alleviate boredom.

### Moderating role of cognitive flexibility

The results of the moderation analysis revealing that cognitive flexibility is a moderator that affects the strength of the relationship between boredom and proactive aggression. Previous research has found that individuals with lower psychological flexibility were more likely to experience depression, anxiety, or worry, while those with higher psychological flexibility had better mental wellbeing since they could choose the right coping mechanisms to adapt to novel situations better (Dawson and Golijani-Moghaddam, 2020). It has been shown that people with a high level of cognitive flexibility are more likely to be able to cope with the COVID-19 epidemic environment than individuals with a low level of cognitive flexibility. Through cognitive restructuring and effective coping, cognitive flexibility might compensate for intolerance of uncertainty*’*s negative impact on psychological well-being. Thus, people with high cognitive flexibility are able to resist behaviors that are harmful to their physical and mental health during the COVID-19 epidemic (Demirtaş, 2021; Sadler et al., 2021).

The results of our study indicate that there was significant negative association between the cognitive flexibility and aggressive behavior. Being high in cognitive flexibility dampens the effect of boredom on aggression. For individuals with high cognitive flexibility, increased boredom did not increase the likelihood of the emergence of individuals’ aggressive behavior. Although aggressive behavior can increase positive emotions, its modulating effect on emotion may only be temporary (Chester et al., 2019). The antisocial nature of aggression dictates that aggression for self-interest and pleasure is inherently contrary to social norms such as morality and law. Individuals may fear poor social evaluation or legal punishment after their aggressive behavior. The duration of pleasure from aggression is relatively short compared to the negative effects of aggression (Miller and Lynam, 2006). In general, aggression is more of a “double-edged sword.” This implies that for individuals with high cognitive flexibility, the use of antisocial behavior such as aggression to regulate emotions is distinctly non-adaptive.

During the COVID-19, closed-off management of the university may contribute to an increased risk of psychological and behavioral problems among college students (Chang and Hou, 2022). Adapting to the restrictive and isolating conditions requires a reappraisal and restructuring of cognitive processes. Since cognitive flexibility provides adaptive solutions to changing conditions and demands, adjustment to this changed context can be particularly difficult for individuals with lower cognitive flexibility. The results of this study indicate that individuals with low cognitive flexibility are more susceptible to boredom levels during closed-off management. The relationship between boredom and aggression varied among individuals who exhibited certain aspects of cognitive flexibility. As boredom increased, proactive aggressive behavior increased for those with low CFI-Alternatives.

As a result of closed-off management, many of the original methods of regulating emotions are limited. In the past, people with low cognitive flexibility might have been able to regulate boredom through activities such as exercise, concerts, and excursions (Tu et al., 2022). It is, however, not possible to obtain these at this time. For people with low CFI-Alternatives, coming up with more solutions is difficult. Proactive aggression that is proactive increases the individual*’*s level of arousal and draws the attention of others. When compared to people with high CFI-Alternatives, they are more likely end up choosing to commit proactive aggression due to a greater focus on short-term positive emotional experiences (Garivani et al., 2021; Kerekes, 2021; Scheinost et al., 2021). Furthermore, although positive emotions do not trigger aggressive behavior (Burgdorf and Panksepp, 2006), the pleasurable experience and the rapid high arousal of aggression may also be an important factor in triggering aggression (Ramírez et al., 2005; Roberton et al., 2012). Individuals may release stress and psychological discomfort by aggressive behavior (Larsen, 2000; Raine et al., 2006). Despite this, for participants with low CFI-Control, two forms of aggressive behavior did not increase with boredom. This is may because people with low CFI-Control engage in less constructive cognition (e.g., wishful thinking or ruminative self-blame) in difficult situations rather than more constructive cognition (e.g., problem solving)(Dennis and Vander Wal, 2010; Lambert et al., 2014; Eadeh et al., 2017).

## Limitations and further work

This study has several limitations, which also provide avenues for future research. Since our study is non-experimental and cross-sectional, we cannot draw causal conclusions from our moderation model. It does not fully account for the causal relationship between aggressive behavior and state boredom in nature, and similar problems exist in studies of aggression with other variables. In light of this, it is necessary to exercise caution when interpreting and extending the conclusions. To address this limitation, future research can use empirical sampling. For example, researchers can ask participants to keep diaries or report their boredom levels at random points over time (Nett et al., 2011).

Although the aggression could regulate emotions, providing pleasure (Raine, 2018). Over time, the individual may become dependent on the aggressive behavior, aggression may be reinforced. Our study further highlights the critical value of enhanced cognitive flexibility in combating the experience of boredom during the COVID-19 epidemic. Psychological interventions that target the improvement of cognitive flexibility could be utilized to reduce psychological symptoms. For example. Interventions such as positive meditation can help individuals develop the belief that aggression is not a reasonable means of regulating emotions, and help them acquire reasonable methods of emotion regulation.

## Conclusion

Our findings indicate that cognitive flexibility is an important factor affecting the relationship between boredom and the two forms of aggression. The results can increase our understanding of the factors that influence aggressive behavior in closed-off management environments. For individuals with high cognitive flexibility, the relationship between state boredom and proactive aggression was not significant. The relationship between state boredom and proactive aggression was significantly positively correlated for individuals with low cognitive flexibility, especially low substitutability. In addition, cognitive flexibility has no significant moderating effect on the relationship between state boredom and reactive aggression. Due to differences in consideration of alternatives and sense of control, boredom may affect decisions about aggressive behavior differently for individuals with different levels of cognitive flexibility. This suggests that cognitive flexibility should be valued as a protective factor that can reduce aggression during closed-off management period of COVID-19 pandemic management (Denson, 2015).

## Data availability statement

The raw data supporting the conclusions of this article will be made available by the authors, without undue reservation.

## Ethics statement

The studies involving human participants were reviewed and approved by Ethics Institutional Review Board of Shandong Drug and Food Vocational College. The patients/participants provided their written informed consent to participate in this study.

## Author contributions

YL: conceptualization, formal analysis, and writing—original draft. XC: investigation, methodology, and writing—review and editing. All authors contributed to the article and approved the submitted version.

## Conflict of interest

The authors declare that the research was conducted in the absence of any commercial or financial relationships that could be construed as a potential conflict of interest.

## Publisher’s note

All claims expressed in this article are solely those of the authors and do not necessarily represent those of their affiliated organizations, or those of the publisher, the editors and the reviewers. Any product that may be evaluated in this article, or claim that may be made by its manufacturer, is not guaranteed or endorsed by the publisher.
